# The Anterior Branch of the Medial Femoral Cutaneous Nerve Innervates Cutaneous and Deep Surgical Incisions in Total Knee Arthroplasty

**DOI:** 10.3390/jcm13113270

**Published:** 2024-05-31

**Authors:** Siska Bjørn, Thomas Dahl Nielsen, Anne Errboe Jensen, Christian Jessen, Jens Aage Kolsen-Petersen, Bernhard Moriggl, Romed Hoermann, Thomas Fichtner Bendtsen

**Affiliations:** 1 Department of Clinical Medicine, Faculty of Health, Aarhus University, 8200 Aarhus, Denmark; anneerrboejensen@gmail.com (A.E.J.); chijss@rm.dk (C.J.); jenspete@rm.dk (J.A.K.-P.); tfb@dadlnet.dk (T.F.B.); 2 Department of Anesthesiology, Aarhus University Hospital, 8200 Aarhus, Denmark; mail@thomasdahl.eu; 3 Department of Anesthesiology, Horsens Regional Hospital, 8700 Horsens, Denmark; 4 Institute of Clinical and Functional Anatomy, Department of Anatomy, Histology and Embryology, Medical University of Innsbruck, 6020 Innsbruck, Austria; bernhard.moriggl@i-med.ac.at (B.M.); romed.hoermann@i-med.ac.at (R.H.)

**Keywords:** medial femoral cutaneous nerve, total knee arthroplasty, postoperative pain, chronic neuropathic pain

## Abstract

**Background/Objectives:** The intermediate femoral cutaneous nerve (IFCN), the saphenous nerve, and the medial femoral cutaneous nerve (MFCN) innervate the skin of the anteromedial knee region. However, it is unknown whether the MFCN has a deeper innervation. This would be relevant for total knee arthroplasty (TKA) that intersects deeper anteromedial genicular tissue layers. Primary aim: to investigate deeper innervation of the anterior and posterior MFCN branches (MFCN-A and MFCN-P). Secondary aim: to investigate MFCN innervation of the skin covering the anteromedial knee area and medial parapatellar arthrotomy used for TKA. **Methods:** This study consists of (1) a dissection study and (2) unpublished data and post hoc analysis from a randomized controlled double-blinded volunteer trial (EudraCT number: 2020-004942-12). All volunteers received bilateral active IFCN blocks (nerve block round 1) and saphenous nerve blocks (nerve block round 2). In nerve block round 3, all volunteers were allocated to a selective MFCN-A block. **Results:** (1) The MFCN-A consistently innervated deeper structures in the anteromedial knee region in all dissected specimens. No deep innervation from the MFCN-P was observed. (2) Sixteen out of nineteen volunteers had an unanesthetized skin gap in the anteromedial knee area and eleven out of the nineteen volunteers had an unanesthetized gap on the skin covering the medial parapatellar arthrotomy before the active MFCN-A block. The anteromedial knee area and medial parapatellar arthrotomy was completely anesthetized after the MFCN-A block in 75% and 82% of cases, respectively. **Conclusions:** The MFCN-A shows consistent deep innervation in the anteromedial knee region and the area of MFCN-A innervation overlaps the skin area covering the medial parapatellar arthrotomy. Further trials are mandated to investigate whether an MFCN-A block translates into a clinical effect on postoperative pain after total knee arthroplasty or can be used for diagnosis and interventional pain management for chronic neuropathic pain due to damage to the MFCN-A during surgery.

## 1. Introduction

The medial femoral cutaneous nerve (MFCN) plays a major role in the skin innervation of the anteromedial knee region [1,2,3,4]. The MFCN originates from the femoral nerve along with the intermediate femoral cutaneous nerve branches, and together they constitute the anterior femoral cutaneous nerve branches [1,5]. The MFCN divides into an anterior (MFCN-A) and a posterior branch (MFCN-P) at the apex of the femoral triangle or more proximally [4,6,7]. A recent study focusing on the midline skin incision for total knee arthroplasty showed that the MFCN-A innervated the skin incision in approximately half of the cases, and the MFCN-P innervated the midline incision in zero cases [4]. This knowledge is important because branches from the MFCN-A may be damaged during the surgical skin incision, potentially causing chronic neuropathic pain. The study presented a novel selective block of the MFCN-A, which could be used as a target for both diagnosis and interventional treatment of chronic neuropathic pain [4].

The MFCN is described as a ‘skin’ nerve in the literature apart from an older dissection study by Horner and Dellon, who briefly mention that the MFCN innervates the medial retinaculum [8]. However, it is not described how this observation was verified or whether it was consistent [8]. For total knee arthroplasty, the currently most common surgical approach is a midline skin incision followed by a medial parapatellar arthrotomy through the deeper tissue layers [9,10]. Our clinical observation is that a subgroup of patients after total knee arthroplasty complain of pain in the anteromedial knee region despite a distal femoral triangle block in the midthigh anesthetizing the saphenous nerve and the medial vastus nerve. This residual pain could be caused solely by insufficient anesthesia of the midline skin incision or because the MFCN has a deeper innervation in the anteromedial knee region including the medial retinaculum.

We hypothesized that the MFCN-A innervates deeper structures in the anteromedial knee region and that the MFCN-A is the main contributor to the innervation of the area covering the medial parapatellar arthrotomy.

Our objective was to investigate whether the MFCN-A and MFCN-P are solely skin nerve branches as indicated by the name and the literature, or if they have a consistent deeper innervation in the anteromedial knee region. A secondary aim was to investigate the contribution from the MFCN to the innervation of the skin area covering the medial parapatellar arthrotomy used for total knee arthroplasty and to the entire anteromedial knee area.

## 2. Materials and Methods

This study consists of a dissection study and unpublished data from a randomized controlled double-blinded volunteer trial (EudraCT number: 2020-004942-12) and a post hoc analysis performed on data from this trial.

### 2.1. Ultrasound-Guided MFCN-A Block

**Figure 1:**
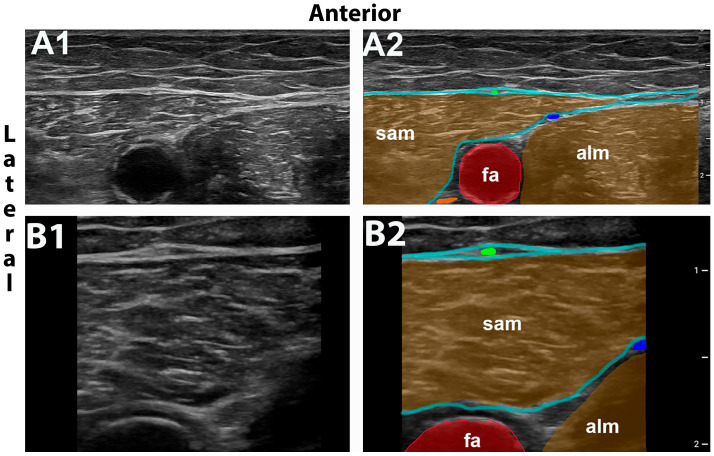
MFCN-A block used in volunteers. Ultrasound images showing the level of injection for the MFCN-A block without (**A1**) and with (**A2**) color overlay using 15 MHz. (**B1**) and (**B2**) is a 19 MHz image without and with color overlay corresponding to (**A1**) and (**A2**). MFCN-A (green dot) can be approached selectively in a duplicature of the fascia lata anterior to the sartorius muscle (sam). Adductor longus muscle, alm; posterior branch of medial femoral cutaneous nerve, blue dot; saphenous nerve, orange dot.

Block technique for the selective MFCN-A block was originally described by Bjørn et al. [4]. The subject is placed supine and the femoral artery identified at the level of the inguinal crease and followed distally. The needle insertion point for the selective MFCN-A block is approximately at the level of the apex of the femoral triangle. Depending on the level of optimal visualization the block can be performed where the MFCN-A is centered on top of the sartorius in cross-sectional view or slightly more distal towards the anterolateral border of the muscle. The MFCN-A descends across the anterior side of the sartorius muscle embedded in a duplicature of the fascia lata (Figure 1) [4].

**Figure 2:**
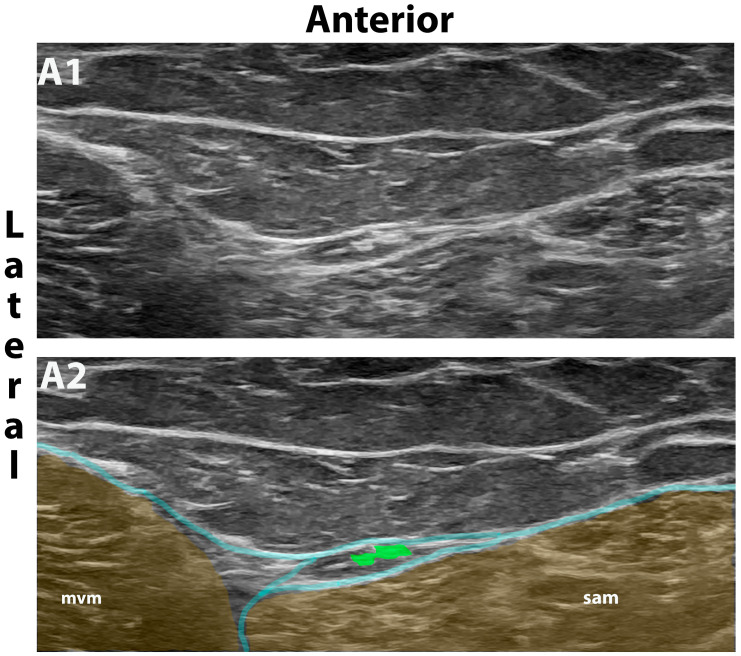
Distal MFCN-A block used in cadavers. Ultrasound image without (**A1**) and with (**A2**) color overlay showing a more distal approach to the selective MFCN-A block compared to Figure 1. The MFCN-A (green dots) is often more easily seen at this distal level approximately at the level of the base of the patella and was therefore used in cadavers where visualization was difficult. The distal approach is not advisable in patients because the MFCN-A has often split into several branches at this level. Mvm, medial vastus muscle; sam, sartorius muscle; fascia lata, cyan lines.

In the dissection study, the selective MFCN-A block was performed at a more distal level [11]. The MFCN-A is often more easily seen approximately at the level of the base of the patella, and therefore this distal approach was used in cadavers where ultrasound visualization is notoriously difficult (Figure 2). In the volunteer study, the more proximal approach described above was applied in order to anesthetize all branches from the MFCN-A [4].

### 2.2. Dissection Study

The dissection study was performed on 16 intact cadaver sides from 8 cadavers donated to the Institute of Clinical and Functional Anatomy of the Medical University of Innsbruck for scientific and educational purposes. In the supine cadaver, the MFCN-A was identified using ultrasound (Figure 2) and marked by injection of 0.1 mL high viscosity special dye.

All injections were performed by the authors Nielsen and Bendtsen. Subsequently, standardized dissections were performed by two other authors, Moriggl and Hoermann. The outcomes of the dissection study were (A) correct identification of the MFCN-A by ultrasound (see below) and (B) MFCN-A and MFCN-P innervation of deeper structures (i.e., deep to the fascia lata) in the anteromedial knee region by dissection. (A): Decision of ‘Correct identification of the MFCN-A by ultrasound’ required that the MFCN-A was correctly stained by injected dye, and that correct identity of the stained MFCN-A was verified by tracing it back to the origin from the MFCN and further to the origin from the femoral nerve during dissection. Meticulous care was taken to separate the MFCN-A from the intermediate femoral cutaneous nerve during dissection. (B): Verification of innervation from the MFCN-A and MFCN-P to deeper structures (i.e., deep to the fascia lata) required that terminal branches from the MFCN-A and MFNC-P could be followed distally and seen to dive into the medial retinaculum.

### 2.3. Volunteer Study

The trial included 20 healthy volunteers of 18 years or older and American Society of Anesthesiology (ASA) I and II classification. Exclusion criteria were inability to cooperate, weight < 60 kg (to avoid local anesthetic systemic toxicity), body mass index > 28, pregnancy, allergy towards local anesthetics, daily use of medicine except oral contraceptives, infection around injection sites and lower limb neuropathy. Lower limb neuropathy was excluded if the volunteer reported no chronic pain in the lower limb and demonstrated normal sensation to pinprick in the dermatomes L2, L3, L4, L5 and S1. The study was conducted on the 12 and 13 December 2020 at the Department of Day Surgery, Aarhus University Hospital.

**Figure 3:**
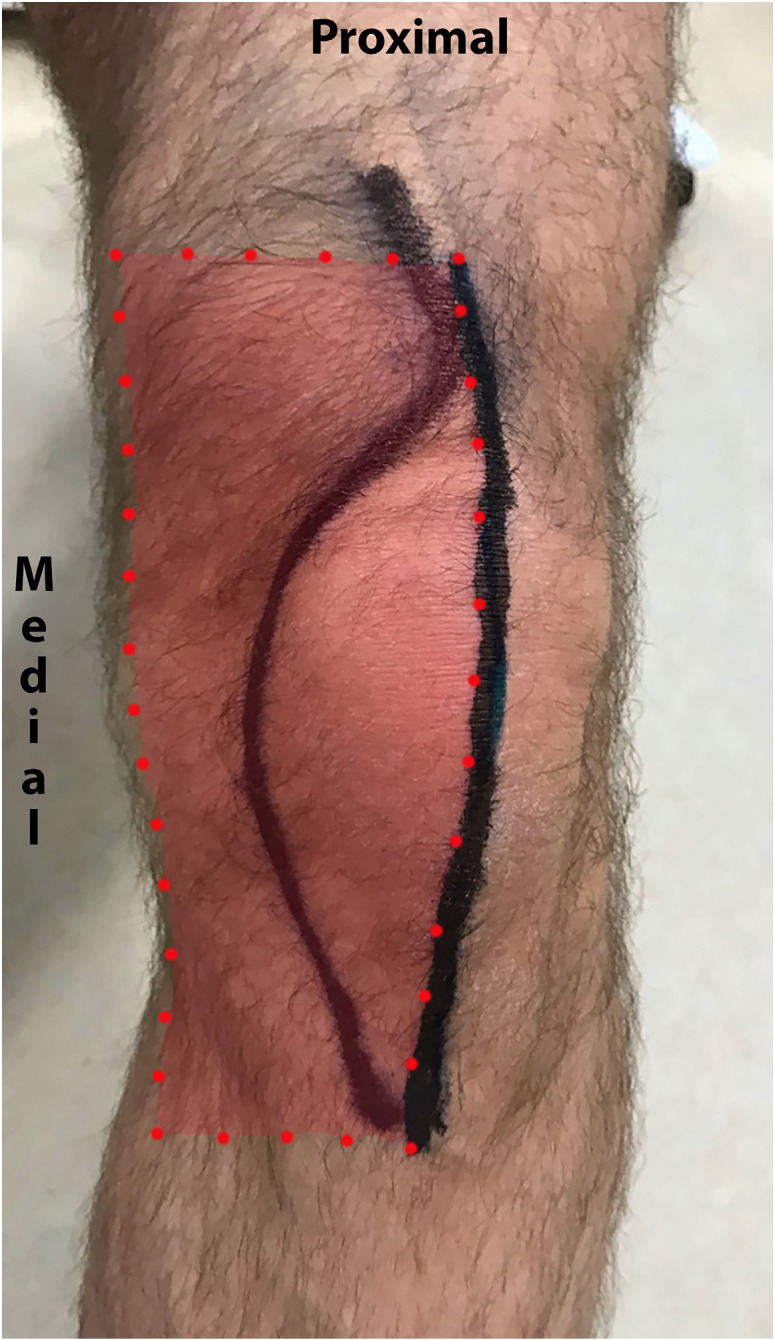
The figure shows a left leg where the midline skin incision and a curved line corresponding to a parapatellar arthrotomy are drawn on the skin. Of note, the parapatellar arthrotomy extends proximal to the midline incision and therefore proximal to the border of the anteromedial knee area shown in red. The anteromedial knee area is defined by the midline skin incision and a vertical line through the medial epicondyle (vertical red stippled line). Note that the parapatellar arthrotomy was drawn per protocol whereas the anteromedial knee area was estimated as a post hoc analysis.

With a skin marker, a standard midline skin incision and a medial parapatellar arthrotomy were drawn bilaterally in all volunteers (Figure 3). Of note, the medial parapatellar arthrotomy exclusively transects tissue layers deep to the skin, but it was drawn on the skin as a visual proxy marker (Figure 3).

**Figure 4:**
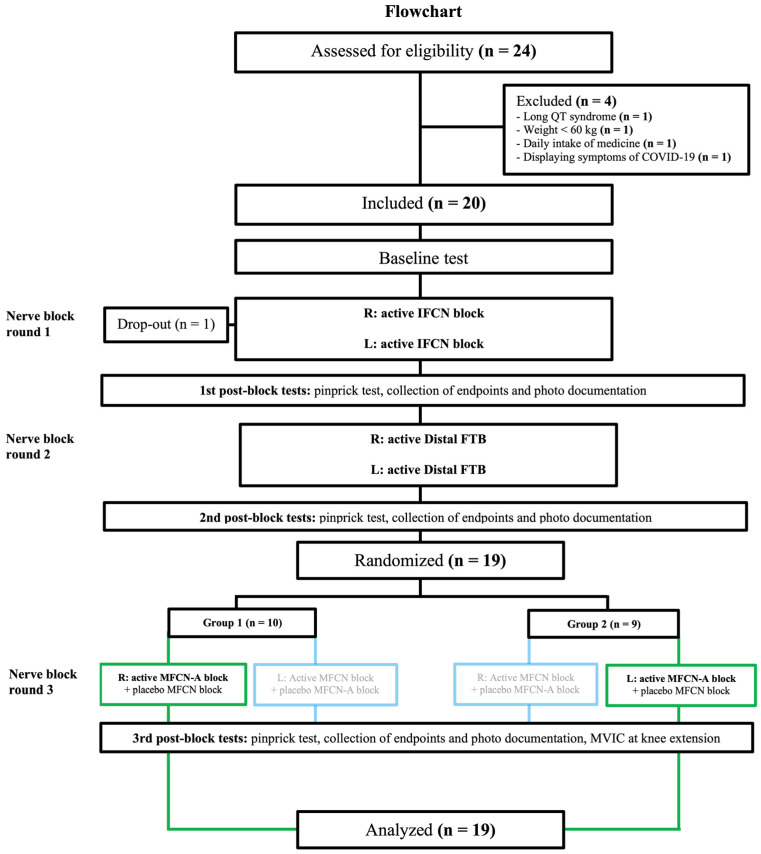
Flowchart. This flowchart presents the design of the nerve block rounds 1, 2 and 3. The original study included a fourth nerve block round, which is not relevant for the present study and not shown [4]. Nerve block rounds 1 and 2 only include active bilateral blocks, as the purpose was to find the subgroup with a non-anesthetized anteromedial skin gap after intermediate femoral cutaneous nerve and saphenous nerve blocks. In nerve block round 3, volunteers received an active block of the MFCN-A on one side (green boxes, bold text). This enabled analysis of how often the MFCN-A anesthetized the skin gap on the anteromedial knee area including the skin area corresponding to the parapatellar arthrotomy. Therefore, this side was the only relevant side for the present post hoc analysis (green boxes, bold text). In the original study the active block of the MFCN-A was combined with a placebo block of both the anterior and posterior branches from the MFCN on the same side (MFCN block) and vice versa on the other side (blue boxes, faded text); however, this was not relevant for the present post hoc analysis. Active indicates bupivacaine 0.25% with epinephrine 1:200,000; placebo indicates saline. FTB: femoral triangle block; IFCN block: intermediate femoral cutaneous nerve block; L: left leg; MFCN block: medial femoral cutaneous nerve block (block of both the anterior and posterior branch); MFCN-A block: selective block of the anterior branch from the MFCN; R: right leg.

Drawing of the parapatellar arthrotomy was described per protocol in the original study; however, these data have not been published previously [4]. In the volunteer trial, active nerve blocks were performed with 0.25% bupivacaine with 1:200,000 epinephrine [4]. All volunteers received bilateral active intermediate femoral cutaneous nerve blocks in the first block round (7 mL) and active bilateral distal femoral triangle blocks in the second block round (10 mL) (Figure 4).

For the intermediate femoral cutaneous nerve block (nerve block round 1) the transducer was placed transversely on the proximal anterior thigh. The intermediate femoral cutaneous nerves (typically two main branches) were targeted where they pierce the fascia lata. They are seen ultrasonographically in a duplicature of the fascia lata or in the subcutis [4]. This is the level of the proximal femoral triangle where the femoral artery has just dived deep to the medial border of the sartorius muscle.

The distal femoral triangle block (nerve block round 2) was performed as previously described [1,4]. The apex of the femoral triangle was identified ultrasonographically where the medial border of the sartorius muscle crosses the medial border of the adductor longus muscle. The needle insertion point was approximately 3–5 cm proximal to the apex of the femoral triangle and the target was the saphenous nerve located anterolateral to the femoral artery.

The intermediate femoral cutaneous nerves innervate the skin covering the anteromedial aspect of the proximal two-thirds of the thigh [1,2]. The infrapatellar branch of the saphenous nerve typically innervates the proximal anteromedial aspect of the lower leg. A distal femoral triangle block at the midthigh level anesthetizes the saphenous nerve including its infrapatellar branch [1,4]. Therefore, the purpose of the first two nerve block rounds was to identify the subgroup of volunteers with a non-anesthetized gap on the parapatellar arthrotomy and in the anteromedial knee area after combined intermediate femoral cutaneous nerve and saphenous nerve blocks.

Subsequently, in the third block round, all volunteers were randomly allocated to an active selective block of MFCN-A (5 mL) on one side (right or left leg) and a placebo MFCN-A block on the contralateral leg (Figure 4). This allowed for estimation of how often the MFCN-A anesthetizes the gap. The randomization only served the purpose of blinding the investigators with no intention of intergroup comparisons.

Thirty minutes after each block round, pinprick tests were performed using a sterile neurological examination pen (Neuropen, Owen Mumford, Woodstock, UK) to define the anesthetized areas on the skin. The first points where the volunteer felt normal, sharp sensation were marked as the circumference of the anesthetized area. All examinations were performed under strict blinding marking the anesthetized areas on the skin with ink only visible in ultraviolet light (i.e., ultraviolet pens), ensuring blinding of the investigator performing the next sensory test [4].

The ‘anteromedial knee area’ was defined as the area between the midline skin incision and a parallel line through the medial femoral epicondyle. The proximal and distal borders was defined by horizontal lines through the most proximal and distal points on the midline skin incision (Figure 3) [1].

The primary study group for outcomes A and C (see below) was the subgroup of volunteers who had a non-anesthetized gap in the anteromedial knee area after intermediate femoral cutaneous nerve and distal femoral triangle blocks (i.e., after the first two block rounds).

The outcomes were as follows: (A) Anesthesia of the medial parapatellar arthrotomy after MFCN-A block; (B) Frequencies of contribution to anesthesia of the medial parapatellar arthrotomy by MFCN-A block, distal femoral triangle block, and intermediate femoral cutaneous nerve block; (C) Anesthesia of the anteromedial knee area after MFCN-A block.

### 2.4. Statistics

Only descriptive statistics were applied for this analysis.

## 3. Results

### 3.1. Dissection Study

In three out of sixteen cadaver sides, dissection of the terminal part of the MFCN-A was not possible due to the condition of the cadaver. In all 13 dissected specimens, it was verified that the MFCN-A had been correctly identified sonographically and marked by ultrasound-guided injection of a special dye.

**Figure 5:**
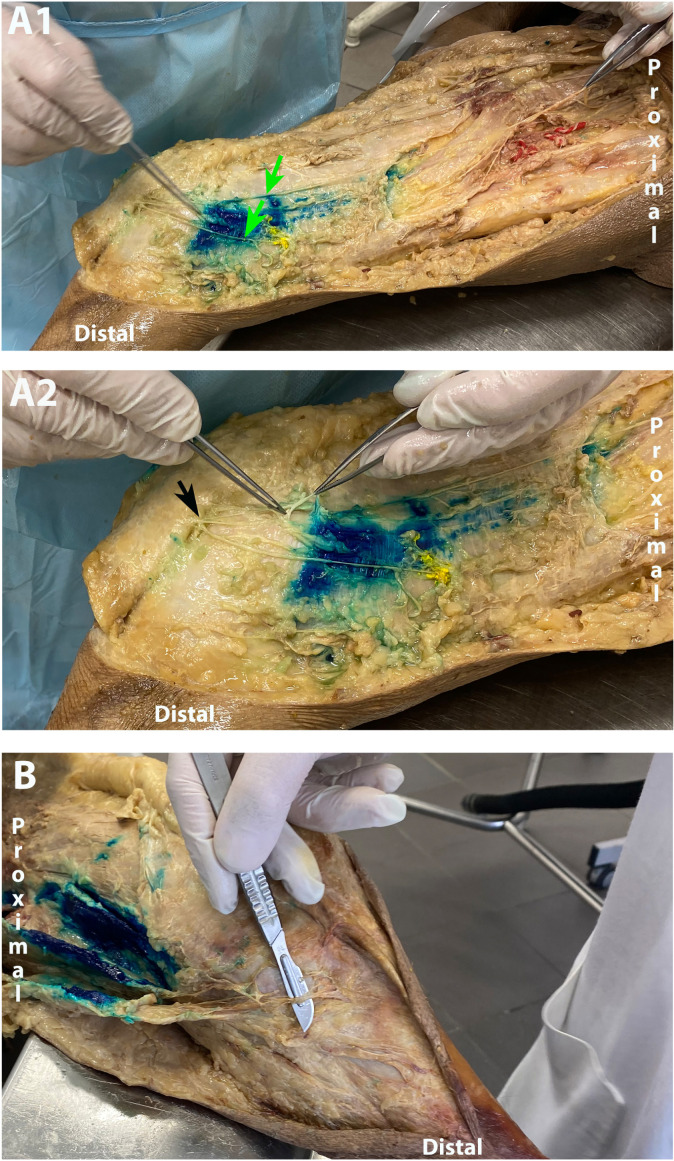
Dissection of the anteromedial knee region. (**A1**,**A2**) show the right side from the same cadaver. In (**A1**), the MFCN-A (lifted by tweezers) gives off minor nerve twigs before splitting into two major branches (green arrows). One of these branches is marked by special dye injection (yellow). The reason that all branches were not targeted was due to the more distal approach used in cadavers (Figure 2). In (**A2**), deep branches from MFCN-A are lifted by tweezers. MFCN-A is also seen to give cutaneous branches (**A2**, black arrow). (**B**) shows the left side from a different cadaver where deep branches from the MFCN-A are indicated by the scalpel. The injection of methylene blue was part of another dissection study and is not related to data captured for the present study.

In 100% of specimens (13/13), it was verified that the MFCN-A branches innervated deeper structures (i.e., deep to the fascia lata) including the medial retinaculum in the anteromedial knee region (Figure 5(A2,B)). The MFCN-P exclusively innervated the skin in the most postero-medial part of the anteromedial knee area or posterior to this area in all 13 specimens with no deep innervation observed.

In eleven specimens, the point of injection was proximal to any major bifurcations of the MFCN-A. In two cases, the MFCN-A bifurcated into two large branches proximal to the point of injection, and only one of the two branches was marked by special dye (Figure 5(A1)). In all 13 specimens, several branches from the MFCN-A were consistently seen to branch off proximal to the point of injection.

### 3.2. Volunteer Study

Twenty volunteers were enrolled, and nineteen volunteers completed the study [4]. Demographics are depicted in Table 1.

**Figure 6:**
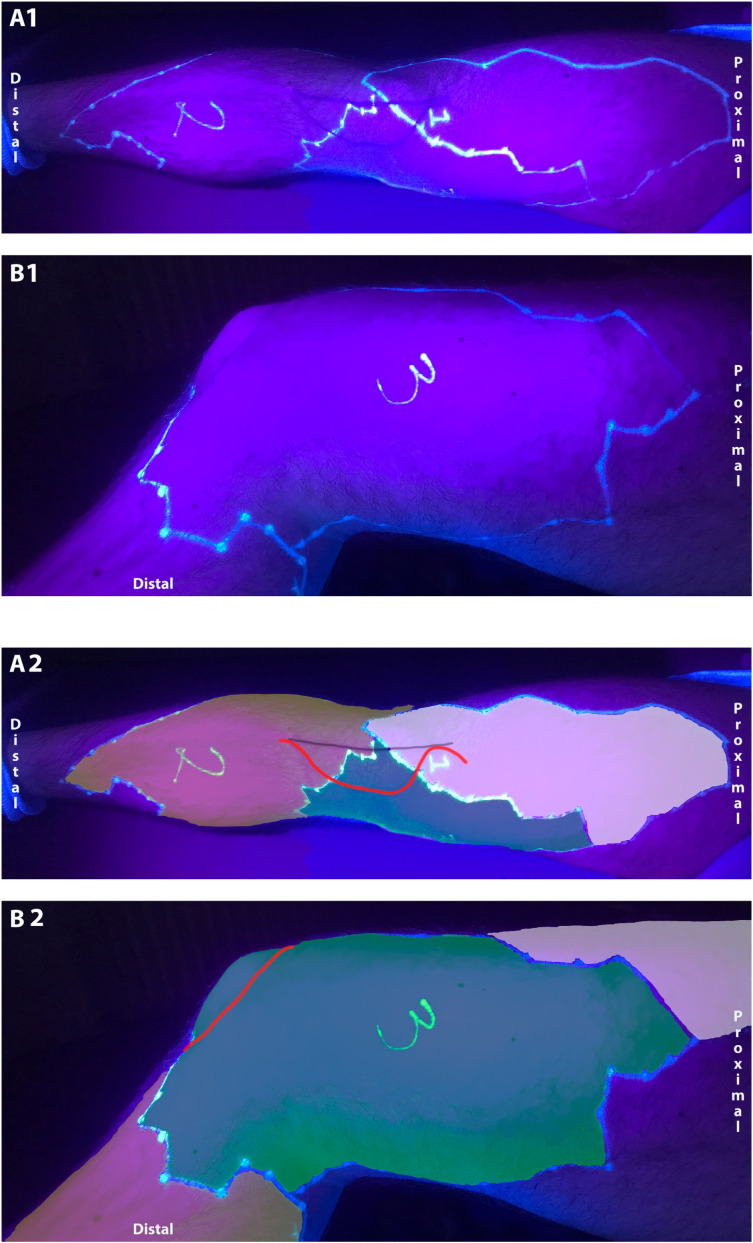
Cutaneous innervation areas. The figure shows a typical example of the cutaneous innervation of the anteromedial knee region in a volunteer seen in anterior (**A1**,**A2**) and medial view (**B1**,**B2**) without (**A1**,**B1**) and with color overlay (**A2**,**B2**). The picture is taken under ultraviolet light because the anesthetized areas were drawn with UV pens invisible to the naked eye. The anesthetized areas after intermediate femoral cutaneous nerve block (light lilac area, #1), distal femoral triangle block (saphenous nerve territory, orange area, #2) and MFCN-A block (green area, #3) are seen in both views. The midline skin incision and medial parapatellar arthrotomy are seen as a black and a red line, respectively (**A2**).

An example of a typical innervation pattern can be seen in Figure 6.

A non-anesthetized gap of the skin covering the medial parapatellar arthrotomy was present after intermediate femoral cutaneous nerve and saphenous nerve blocks in 11 out of the 19 volunteer legs (58%) allocated to receive an active MFCN-A block in block round 3 (Figure 4). The frequency of complete anesthesia of the gap after the MFCN-A block was 9 out of 11 (82%).

The frequencies of contribution to the innervation of the skin covering the medial parapatellar arthrotomy were as follows: MFCN-A 9/19 cases (47%), intermediate femoral cutaneous nerve 19/19 cases (100%), and saphenous nerve 16/19 cases (84%).

A non-anesthetized gap of the skin in the anteromedial knee region was present after intermediate femoral cutaneous nerve and saphenous nerve blocks in 16 out of the 19 volunteer legs (84%) allocated to receive an active MFCN-A block in block round 3 (Figure 4). The frequency of complete anesthesia of the gap after the MFCN-A block was 12 out of 16 (75%).

## 4. Discussion

The results from the dissection study show that the MFCN-A consistently innervates genicular layers deep to the skin in the anteromedial knee region including the medial retinaculum. This is an interesting finding because the MFCN-A is described as an exclusively cutaneous nerve in the literature apart from a brief mentioning of an innervation to the medial retinaculum by Horner and Dellon [8]. The deep innervation from the MFCN-A makes it relevant in relation to medial parapatellar arthrotomy. The fact that the MFCN-A is not only a cutaneous nerve but also innervates deeper structures in a large area of the anteromedial knee region makes it potentially interesting in relation to acute postoperative pain in the anteromedial knee area. Furthermore, deep branches from the MFCN-A may be injured during medial parapatellar arthrotomy and could therefore potentially cause neuropathic pain after total knee arthroplasty.

No deep innervation by the MFCN-P in the anteromedial knee region was observed during dissection in any cadavers. Thus, MFCN-P innervation of the skin covering the medial parapatellar arthrotomy is not a relevant proxy marker of deep innervation, and no risk of intersection of MFCN-P branches during a medial parapatellar arthrotomy would be present.

The results from the volunteer trial show a high frequency of incomplete anesthesia of the anteromedial knee region including the medial parapatellar arthrotomy after an intermediate femoral cutaneous nerve block and a distal femoral triangle (i.e., saphenous nerve) block. The addition of an MFCN-A block in the third block round effectively anesthetized the skin in the anteromedial knee region including the skin over the medial parapatellar arthrotomy in most cases. The fact that the success rate of complete cutaneous coverage of the gap after a selective MFCN-A block is high shows that the MFCN-A and not the MFCN-P is the important branch for complete cutaneous anesthesia of medial parapatellar arthrotomy and the anteromedial knee area. The dissection showed a consistent deep innervation from the MFCN-A and not the MFCN-P in the anteromedial knee area in all cadavers. This suggests that the MFCN-A could potentially be accountable for acute nociceptive pain and chronic neuropathic pain in the anteromedial knee region after total knee arthroplasty including the area of the medial parapatellar arthrotomy.

Based on our results from dissection and a volunteer trial, it is not possible to assess whether the deep MFCN-A innervation of the anteromedial knee region translates into a clinical effect in relation to acute pain after total knee arthroplasty. Very few studies have investigated the effect of a selective block of the MFCN on acute pain after total knee arthroplasty. Kampitak et al. showed that a selective block of the anterior femoral cutaneous nerve branches (i.e., an intermediate femoral cutaneous nerve block and MFCN block) combined with a distal femoral triangle block significantly improved acute pain relief after total knee arthroplasty compared to a standalone distal femoral triangle block [12,13]. Furthermore, several clinical studies have compared an injection lateral to the femoral artery at the level where the medial border of the sartorius muscle first covers the femoral artery (i.e., in the proximal femoral triangle) to an injection at the midthigh level midway between the anterior superior iliac spine and the base of the patella (distal femoral triangle block) [14,15,16,17]. The difference between the proximal and distal approach to the femoral triangle block is that the MFCN may be anesthetized by the proximal approach. Two of the studies observed superior pain relief in the group receiving a proximal femoral triangle block after total knee arthroplasty and anterior cruciate ligament reconstruction, respectively [16,17]. The studies detecting no difference between a proximal and distal femoral triangle block in relation to acute pain after total knee arthroplasty were powered to detect only very large reductions in opioid consumption [16,17]. The results showed a clear tendency of improved pain relief in the proximal group compared to the distal; however, the difference did not reach statistical significance [16,17]. Importantly, the MFCN was not specifically targeted during the proximal approach in any of the cited studies which may also have reduced the intergroup difference in postoperative pain [14,15,16,17]. Thus, clinical trials investigating the effect of supplemental anesthesia of the MFCN-A on pain after total knee arthroplasty are needed. In our clinical experience, a large subgroup of patients complains of acute pain in the anteromedial knee region after total knee arthroplasty despite a successful combined femoral triangle block and intermediate femoral cutaneous nerve block. This residual pain is typically relieved by the selective MFCN-A block. In addition, we have identified a large group of patients with chronic neuropathic pain in the anteromedial knee region allocated to our outpatient nerve injury clinic. This chronic neuropathic pain is often relieved by a diagnostic selective MFCN-A block, which identifies MFCN-A as a target of interventional pain management.

The main limitation of this study is that it is primarily based on cadaver dissection and healthy volunteers, and therefore the relevance in the clinical setting remains to be clarified. Future studies on patients undergoing total knee arthroplasty are needed to investigate the importance of the MFCN-A in the clinical setting. Another limitation is that part of the volunteer study is a post hoc analysis. Two of the outcomes (A and B) were, however, prospectively planned per protocol. Furthermore, it is a limitation that the anesthesia of the medial parapatellar arthrotomy was assessed by anesthesia of the congruent skin territory as a proxy marker in the healthy volunteers. However, the dissection study demonstrated consistent MFCN-A innervation to the deep layers in the anteromedial knee region.

## 5. Conclusions

In conclusion, our results from the dissection showed that the MFCN-A consistently innervates deeper structures in the anteromedial knee region including the medial retinaculum. Furthermore, results from our volunteer study showed that the anteromedial knee region including the skin area covering the medial parapatellar arthrotomy for total knee arthroplasty is incompletely anesthetized by the combined intermediate femoral cutaneous nerve and saphenous nerve blocks in the majority of cases. An additional MFCN-A block anesthetizes this area with a high success rate. Future clinical trials are needed to investigate whether anesthesia of the MFCN-A is important for relief of acute pain after total knee arthroplasty. Furthermore, the MFCN-A is at high risk of transection not only during the midline skin incision, but also during the parapatellar arthrotomy used for total knee arthroplasty due to the deep innervation. The MFCN-A may therefore potentially be involved in the development of chronic neuropathic pain after total knee arthroplasty.

## Figures and Tables

**Table 1:** Demographics of the volunteers who completed the study.

Number of volunteers	19
Age, mean (SD)	23.6 (2.8) years
Sex, n (%)	
Male	13 (68%)
Female	6 (32%)
ASA status, n (%)	
ASA I	19 (100%)
ASA II	0 (0%)
Height, mean (SD)	180.0 (9.2) cm
Weight, mean (SD)	75.3 (9.5) kg
BMI, mean (SD)	23.2 (1.9)

## Data Availability

The data presented in this study are available on request from the corresponding author. The data are not publicly available in order to maintain participant privacy.
